# The Impact of the Metabolic Syndrome Severity on the Appearance of Primary and Permanent DNA Damage

**DOI:** 10.3390/medicina61010021

**Published:** 2024-12-27

**Authors:** Mirta Milić, Luka Kazensky, Martina Matovinović

**Affiliations:** 1 Division of Toxicology, Institute for Medical Research and Occupational Health, Ksaverska cesta 2, 10000 Zagreb, Croatia; lkazensky@imi.hr; 2 Department of Internal Medicine, Division of Endocrinology, University Hospital Centre Zagreb, Croatian Referral Center for Obesity Treatment, Kišpatićeva 12, 10000 Zagreb, Croatia; martina_10000@yahoo.com; 3 The Faculty of Kinesiology, University of Zagreb, Horvaćanski zavoj 15, 10000 Zagreb, Croatia

**Keywords:** alkaline comet assay, micronucleus cytome assay, DNA repair

## Abstract

The prevalence of metabolic syndrome (MetS) worldwide is rapid and significant on a global scale. A 2022 meta-analysis of data from 28 million individuals revealed a global prevalence of 45.1%, with notably higher rates in the Eastern Mediterranean Region and the Americas, particularly in high-income countries. MetS is associated with impaired antioxidant defense mechanisms, resulting in the excessive generation of reactive oxygen and nitrogen species (RONS) and elevated levels of DNA damage. Unrepaired damage can lead to DNA base changes, chromosomal mutations, genomic loss and instability, and disrupted gene and protein expression. Such changes contribute to an increased risk of tumorigenesis, cancer progression, and mortality. The alkaline comet and micronucleus *cytome* assay are commonly used assays for DNA damage evaluation. The estimation of damage with those two techniques demonstrated the link between the increased risk of cancer and mortality. Incorporating these techniques in a set of biomarkers to assess the MetS severity holds promise; however, comprehensive literature reviews featuring large-scale studies integrating both assays remain scarce. This systematic review aims to integrate and critically evaluate the existing scientific literature regarding this topic.

## 1. Introduction

Before the onset of metabolic syndrome (MetS), various metabolic derangements are often already present, usually linked to adiposity. Adiposity itself is frequently a consequence of overnutrition combined with sedentary lifestyle. Once MetS is developed, it increases the risk of developing type 2 diabetes (T2D) [1,2,3] and cardiovascular diseases (CVDs) [1,4], as well as the likelihood of premature mortality [1,4,5]. As a result, MetS presents a major global health issue and calls for significant public health intervention.

Although substantial literature on MetS and its components exists, the first meta-analysis encompassing global data from 28 million individuals was only published in 2022. This analysis demonstrated the high global prevalence of MetS and its components in the adult population, while also highlighting the growing concern of MetS prevalence in children and adolescents [1]. The meta-analysis further revealed considerable variability in the prevalence of MetS components globally, attributable to differences in diagnostic MetS definitions and characterization criteria [1] (for criteria differences, see Table 1).

MetS is broadly defined as a cluster of metabolic conditions, including hypertriglyceridemia, hyper-low-density lipoproteins, hypo-high-density lipoproteins, insulin resistance, abnormal glucose tolerance, and hypertension. Although several international organizations provide varying diagnostic criteria for MetS, they generally agree that a diagnosis requires the presence of at least three of the following traits: elevated waist circumference, high blood pressure, high triglyceride levels, elevated fasting blood glucose, and/or reduced high-density lipoprotein levels (see Table 1 for a detailed criteria comparison).

A proinflammatory state usually accompanies MetS. Chronic inflammation, driven by an imbalance between heightened inflammatory stimuli and reduced anti-inflammatory mechanisms, is thought to be a key contributor to MetS pathogenesis [8]. Persistent low-grade inflammation is characterized by the hypersecretion of proinflammatory cytokines, which can result from resistance to insulin’s anti-inflammatory effects in genetically or metabolically predisposed individuals, as well as from factors such as overnutrition, physical inactivity, aging, and excess adiposity—all of which are features of MetS [8]. An increased inflammatory state is also linked to increased levels of reactive oxygen (ROS) and nitrogen species (RONS) [9,10], leading to elevated levels of oxidative stress (OS). Excessive ROS quantities without adequate removal pose a significant challenge to maintaining normal cellular and systemic function [10].

Adipose tissue is a significant source of ROS production. Therefore, OS can play a causal role in obesity by promoting pre-adipocyte proliferation, differentiation, the expansion of white adipose tissue, and altered food intake [10]. It can increase the production of cytokines such as tumor necrosis factor-alpha, generating more ROS and worsening lipid peroxidation [10]. OS can also increase the release of interleukins from immune cells and adipocytes, and reduce systemic anti-inflammatory cytokines, by promoting systemic inflammation [8]. ROS targets mainly lipids and other macromolecules, causing their peroxidation and accumulation of inactive proteins [10]. This disrupts redox signaling to promote proinflammatory and pro-fibrotic pathways, which affect insulin metabolic signaling and endothelial dysfunction, promoting further target organ damage, mostly cardiovascular and renal [10]. Altered lipid and glucose metabolism (including glucose auto-oxidation and non-enzymatic glycation), chronic inflammation, tissue dysfunction, and ROS formation increase OS further in obese people [10]. There is evidence that MetS patients exhibit elevated levels of mitochondrial OS and dysfunction, which are the primary causes of oxidative damage and metabolic abnormalities [10].

Oxidative stress and inflammation are also linked to increased levels of DNA damage, genomic instability, and an increased risk of cancer [9]. Persistent DNA damage, if unrepaired, coupled with the inability to control inflammatory responses, can initiate tumorigenesis [9]. Individuals with MetS are at double the risk of cardiovascular mortality and have a 1.5 times the risk of all-cause mortality [11]. Evidence suggests that at least two of five DNA damage repair mechanisms are repressed/inactive among obese and severely obese individuals (with a body mass index higher than 30 or 35 kg m^−2^) [12,13,14]. The measurement of DNA damage using two genotoxic assays with established links to cancer and mortality prognoses offers valuable potential as biomarkers for disease severity estimation, therapeutic efficacy, and comorbidity prevention, including cancer [15,16]. Among these assays, the alkaline comet and micronucleus cytome assays are particularly notable [12,15,16].

**Figure 1:**
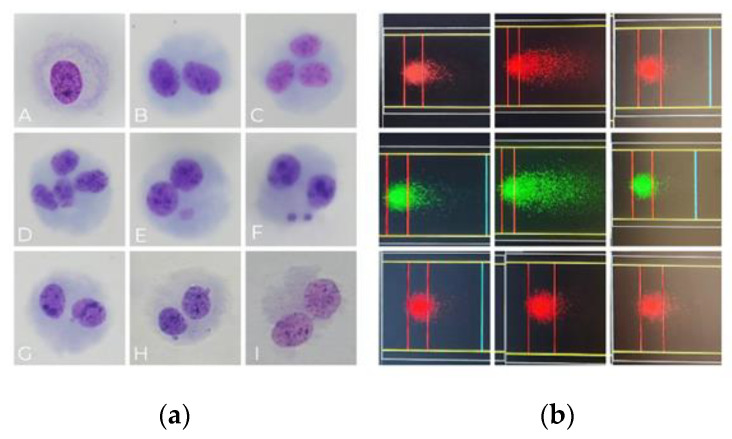
(**a**)—Photomicrographs of the cells scored in the CBMN cytome assay stained with Giemsa, 1000× magnification: (**A**) mononucleated cell, (**B**) binucleated (BN) cell, (**C**) trinucleated cell, (**D**) tetranucleated cell, (**E**) BN cell with MN, (**F**) BN cell with two MNs, and (**G**) BN cell with nuclear bud (NBUD). NBUD represents gene amplification, (**H**) BN cell with two NBUDs, and (**I**) BN cell with nucleoplasmic bridge (NPB). NPB represents bicentric chromosome; (**b**)—different types of damaged (with tail) and non-damaged cells (without the tail) in the alkaline comet, with different filters only for imaging purposes, 400× magnification, captured with Metafer semi-automated microscope for slides and picture analysis.

The alkaline comet assay, a simple and sensitive method for detecting DNA damage (Figure 1), can provide results on the same day as sampling when applied to peripheral blood, eliminating the need for cell culture. The assay detects primary DNA damage by visualizing fragmented DNA migration during electrophoresis, forming a characteristic “comet” shape in agarose gels.

The alkaline comet assay, with modifications incorporating specific enzymes, enables the assessment of various types of DNA, including oxidative DNA damage [17,18,19,20]. For instance, utilizing the formamidopyrimidine DNA glycosylase (Fpg) or 8-oxo-7,8-dihydro-2′-deoxyguanosine (8-oxo-dG), the assay can identify oxidized purines or guanines, respectively, providing insights into specific oxidative damage [19].

With a database encompassing over 19,000 individuals, this technique has facilitated the estimation of DNA damage in diseased individuals, with and without tumors, and in those with a high mortality risk [16,21].

Permanent DNA damage can be propagated to daughter cells during cell division and is measurable using the micronucleus (MN) *cytome* assay. Permanent DNA damage can create a pool of cells with persistent genomic instability, heightening cancer susceptibility. The MN assay often requires establishing cell cultures, as it detects unrepaired DNA damage after the first cell division, during which cells attempt to repair damage. The method identifies persistent DNA damage in the form of micronuclei—small nuclei in the cytoplasm—representing either the loss of chromosomal fragments or whole chromosomes excluded from the mitotic spindle during division (Figure 1).

In the MN assay for human lymphocytes, the cell cultures are maintained for 72 h, with cytochalasin B added at the 44-h mark. This compound inhibits cytokinesis while allowing karyokinesis, resulting in multinucleated cells. This step enables the analysis of permanent DNA damage in the shared cytoplasm of two daughter nuclei. This assay has since evolved into the cytokinesis-block micronucleus *cytome* assay (CBMN), which measures different types of DNA damage through a well-established protocol [22]. Peripheral blood lymphocytes, frequently used for DNA damage studies, retain DNA damage sites for extended periods of time, making them ideal for such analyses [23,24]. A large international cohort study demonstrated a correlation between the MN frequency in healthy subjects and cancer risk, linking the individuals with medium-to-high MN frequencies to increased susceptibility [15].

This review explores the importance of and the association between DNA damage levels detected by the comet and MN assays and MetS occurrence. Individuals with MetS are recognized as a high-risk group for tumorigenesis. Given the direct relationship between genomic instability and tumor incidence, we also evaluated the relevance of measuring DNA damage in MetS. This review identifies gaps in the existing scientific literature that should be assessed to provide deeper insights and determine whether these assays could serve as biomarkers for MetS severity evaluation, and possible prognostic biomarkers of genomic instability and cancer risk. The results of prior studies could support the hypothesis that both MN and comet assays may expand the prognostic power of biomarkers for the detection and progression of MetS to even diabetes mellitus (DM) and cardiovascular disease (CVD). However, no prior reviews have jointly analyzed the use of both assays to establish such connections.

## 2. Materials and Methods

**Figure 2:**
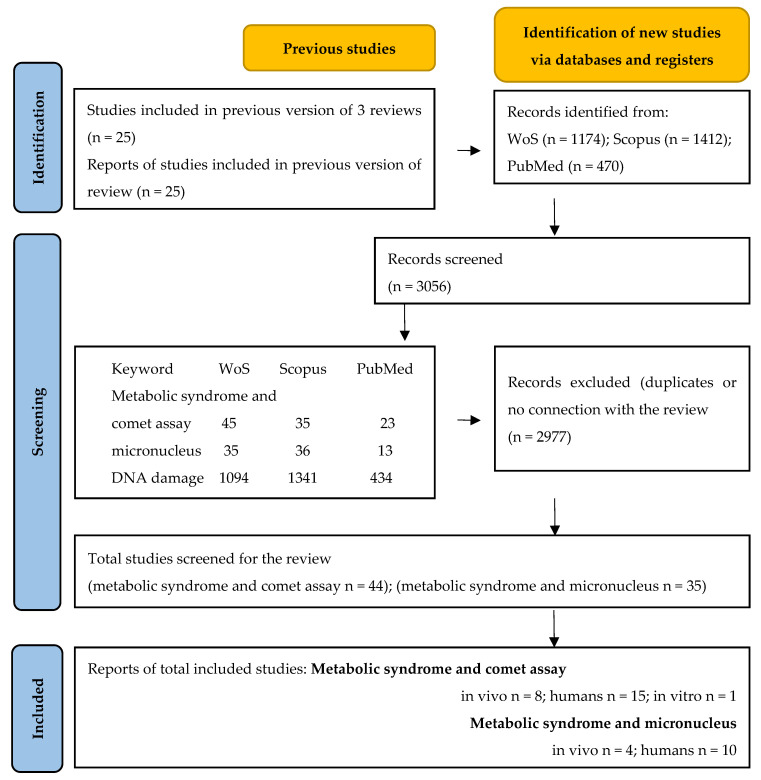
PRISMA 2020 flow diagram for updated systematic reviews which included searches of databases and registers only.

In this review, we adhered to PRISMA guidelines, and a comprehensive checklist is available in the Supplementary Materials. We conducted a search across three major databases, PubMed, Web of Science, and Scopus, focusing on studies in English that examined the comet assay, micronucleus assay, or both, in the context of MetS (human studies, in vitro, in vivo, and ex vivo; for details, see Figure 2).

No time restrictions were imposed, and studies published up to 6 August 2024, were included. Using the keywords “metabolic syndrome”, “comet assay”, “micronucleus”, and “DNA damage”, we identified a total of 3056 articles across these databases, which were screened and assessed (see Figure 2 for details).

Ultimately, 24 studies on the comet assay and 14 on the MN assay met the inclusion criteria. Among these studies, six utilized both assays. For clarity, while these studies were counted separately in the assay-specific totals, the results from each study were consolidated in the manuscript to avoid redundancy, resulting in a total of 32 described studies rather than the sum of the individual assay totals (38).

## 3. Results and Discussion

Our literature search identified three review articles addressing the connection between DNA damage, obesity, MetS, and associated factors of diet and weight [25,26]. These reviews primarily focused on the MN assay, with only one examining the comet assay. Usually, only one MN assay parameter was analyzed, and elevated DNA damage levels were found in individuals with obesity, MetS, and related comorbidities. We will start our explanations with studies on obese and overweight persons.

A global meta-analysis reported central obesity (45.1%) as the most prevalent MetS component, particularly in ethnic-specific populations. Interestingly, a cohort study involving 12,052 males demonstrated lower MN frequency in overweight individuals than in normal-weight males, contrary to most findings [27]. Santovito et al. 2020 demonstrated a correlation between all of the CBMN *cytome* assay parameters with BMI in 150 participants, ranging from normal weight to overweight [28]. Another study, including 83 obese, 21 overweight, and 21 normal-weight subjects, demonstrated significantly higher frequencies of MN, nucleoplasmic bridges (NPB), nuclear buds (NBUD), and apoptotic and necrotic cells in the lymphocytes of obese subjects compared to their normal-weight and overweight counterparts [29]. In addition, the frequencies of all of the mentioned parameters in total overweight/obese and all subjects increased with an increasing BMI. Similar trends were observed in overweight subjects and children with obesity, with a 2.5- to 2.7-fold increase in the MN frequency [30]. In their review, Franzke et al. 2020 [31] conducted a meta-analysis of published data regarding in vivo MN assay human observational studies, and their connection with diabetes and obesity. A meta-analysis was performed on 1658 individuals (183 obese, 528 overweight, and 947 normal-weight subjects). Their results linked obesity and overweight to elevated DNA damage levels (mainly the MN frequency) in adults and children, noting heightened risks for cancer, diabetes, and cardiovascular disease [31].

Gandhi et al., 2012 and Tomasello et al., 2011 demonstrated elevated DNA damage parameters in individuals with obesity using the comet assay [32,33]. Wlodarczyk et al., 2018, found a twofold increase in DNA damage in 88 women with obesity compared to 26 controls, with the BMI and daily food intake being the key predictors [34]. Their data showed that an adequate diet rich in antioxidants such as vitamins C and E could reduce the impact of obesity-associated inflammation on DNA damage. High visceral fat area in men was linked to higher levels of DNA damage as measured with the alkaline comet assay in Jang et al., 2003 [35]. In 300 morbidly obese women, Luperini et al., 2015, demonstrated elevated levels of DNA damage as measured with the comet assay when compared to 300 eutrophic women [36]. Bukhari et al., 2010, also showed elevated levels of comet assay descriptors (tail length) in 160 individuals with obesity compared to 160 lean controls, and a positive correlation of comet assay descriptors with the BMI, TG, LDL, total cholesterol, MDA, and ALT levels [37]. Dos Santos et al., 2019 [38], examined 158 coal miners, with most being overweight or obese, and found a significant positive correlation between BMI and DNA damage as measured with the comet assay, especially among workers with some non-communicable diseases.

It seems that different types of DNA damage occur during different phases of MetS development. We already demonstrated that, in the early stage of MetS, DNA damage assessed with the comet assay was not significantly elevated [39]. Pilar et al., 2014 [40], demonstrated elevated levels of DNA damage assessed by the comet assay in MetS patients with lower levels of MN compared to the control group. Demirbag et al., 2006 [41], demonstrated a significant increase in comet assay descriptors (arbitrary units) among 65 MetS and 65 control patients, while also identifying age, BMI, and components of MetS characterization as independent predictors of DNA comet assay damage in MetS patients. Karaman et al., 2015 [42], examined 52 MetS patients and 35 controls utilizing the MN and comet assay, and demonstrated elevated levels of DNA damage in the MetS group and a positive correlation with the waist circumference, BMI, and plasma triglyceride levels.

Polycystic ovary syndrome (PCOS), a common condition in reproductive-aged women, is associated with impaired glucose tolerance, T2D mellitus, and the MetS. Elevated levels of DNA damage have been reported for this syndrome utilizing both assays [25,43,44]. A study by Zaki et al., 2018 a,b, demonstrated increased levels of DNA damage as evaluated by the comet assay, which was significantly higher in female participants who met the criteria for PCOS diagnosis [44,45]. Elevated levels of DNA damage in the comet assay correlated with the waist circumference. An increased MN frequency was reported in studies by Yesilada et al., 2006, Moran et al., 2008, and Hamurcu et al., 2010 [46,47,48]. These studies also found a positive correlation between increased MN frequencies and chromosome mis-segregation, which were positively correlated with the BMI and insulin resistance levels. It is important to note that obese or overweight women with PCOS have an increased risk of developing endometrial cancer, as highlighted by Soares et al., 2016 [43].

One of the diagnostic criteria for MetS diagnosis criteria is a pre-existing diagnosis of diabetes. There is limited research on type 1 diabetes (T1D) and both DNA damage assays. Cinikilic et al., 2009, utilized the CBMN cytome assay [49], demonstrating elevated levels of MN, nuclear buds, and nucleoplasmic bridges. However, the differences between 35 patients and 15 controls were insignificant, though the assay still revealed the influence of T1D on DNA damage stability. Franzke et al., 2020, conducted a meta-analysis demonstrating that diabetes (both type 1 and type 2) significantly affects DNA stability. Their analysis included 77 T1D patients with 100 controls and 543 T2D patients with 320 controls, highlighting the role of insulin resistance or deficiency in mitochondrial dysfunction and subsequent DNA damage [31].

In T2D, several studies demonstrated significantly elevated levels of MN frequency compared to the controls [50,51] or normoglycemic controls [52]. In 210 patients with ischemic heart disease, diabetes was an independent factor in increased MN frequency (Andreassi et al., 2005, and Andreassi et al., 2011—review) [25,53]. Salimi et al., 2016, examined 200 individuals, showing increased DNA damage in T2D patients, with even higher levels in those with nephropathy [54]. Corbi et al., 2014, studied 150 participants across groups defined by diabetes mellitus (DM), dyslipidemia, and periodontitis (PD) status, finding elevated MN frequencies in DM groups and CBMN *cytome* assay parameters, such as nucleoplasmic bridges [55]. Higher values were also present in the group with only dyslipidemia and PD without DM and in the group with dyslipidemia, PD, and well-contained DM. Deo et al., 2021, conducted a meta-analysis on 456 T2D subjects and 280 controls, demonstrating increased MN frequency in T2D, which increased when comorbidities like periodontitis, dyslipidemia, or cardiovascular disease (CVD) were present [56].

Buccal MN *cytome* assays, a less invasive method, have shown a good correlation with the MN frequency in lymphocytes, with some studies reporting up to twofold increases in T2D patients (see Andreassi et al., 2011, and Setayesh et al., 2019, for details) [12,25]. Six studies have used this assay in T2D, demonstrating significant DNA damage in most cases (see the reviews mentioned above).

The comet assay and its 8-oxo-dG variant have been less helpful as markers in early MetS [39,57], likely due to the selection of younger subjects with robust DNA repair capacities. For instance, 8-oxod-dG levels were higher in normal-weight obese women than in obese women, but alkali labile sites were higher in the latter [33]. These demonstrate that both assays can serve as a prognostic differential indicator between normal-weight obese and obese (higher alkaline labile sites in the latter). Normal-weight obese subjects are those with a normal weight and body mass index but whose fat mass is >30% of their total body weight and whose risk of developing obesity-related diseases is likely increased. Maynard et al., 2015 [58], revealed associations between vitality, BMI, and DNA damage, mostly single-strand breaks and alkali-labile sites, utilizing the alkaline and Fpg-modified alkaline comet assays. Other studies on non-obese people did not demonstrate the connection between BMI and oxidative DNA damage sites [59,60,61]. Tomasello et al., 2011, showed elevated levels in overweight and obese women [33]. In 300 women with morbid obesity, Luperini et al., 2015, demonstrated elevated Fpg levels of DNA damage as measured by the comet assay compared to 300 eutrophic women [36].

Our previous study [26] combined biochemical parameters with all three DNA damage assays (MN, comet, and Fpg-modified alkaline comet), revealing the sequence of disease development. Initially, blood cell counts and cell division alterations appeared, followed by MN assay changes (e.g., NBUDs and NPBs), culminating in decreased cell proliferation and an elevated micronucleus frequency. Elevated primary DNA damage detected by the comet assay was significant in individuals with severe obesity but not overweight ones. These findings highlight the assays’ sensitivity in detecting genomic instability and their potential as biomarkers for obesity-related health risks, comorbidities, cancer predisposition, and a higher mortality risk with the loss of 7–10 disease-free years [62].

The assays can also serve as early indicators in the development of obesity and MetS, even in individuals with a normal or low BMI where oxidative DNA damage is present. These assays are particularly valuable as early clinical biomarkers for diabetic complications. For detailed insights into the mechanisms of DNA damage and obesity, refer to Wlodarczyk et al., 2019 [34]. Premenopausal women with obesity who were diagnosed with two, three, or from three to five MetS components exhibited significantly higher DNA damage levels compared to the controls. Furthermore, those with four to five MetS components had substantially higher DNA damage than individuals with only two components [44]. The assays have also proven helpful in predicting diabetic complications, such as microangiopathy and macroangiopathy [63,64,65,66,67]. Interestingly, the comet assay observed that oxidative DNA damage was higher in individuals with T2D than those with T1D [67]. In a study by Gelaleti et al., 2015 [68], involving 111 pregnant women with diabetes or mild gestational hyperglycemia, elevated levels of DNA damage were identified. This damage type varied according to the glycemic profile and oxidative stress. Pregnant women with diabetes showed increased levels of oxidative DNA damage (oxidized purines), probably due to hyperglycemia. In contrast, women with mild gestational hyperglycemia exhibited oxidative damage (oxidized pyrimidines) associated with obesity, hypertension, and/or insulin resistance.

Finally, weight loss can improve health, decrease the severity of MetS, and improve DNA stability [67,68]. In our previous studies, using all three assays [26], we demonstrated that a 3-week low-calorie diet could improve health and DNA stability in individuals with severe obesity, though levels of DNA damage remained above those of healthy controls. Therefore, these assays provide crucial insights into severe obesity and MetS genomic stability [12].

**Figure 3:**
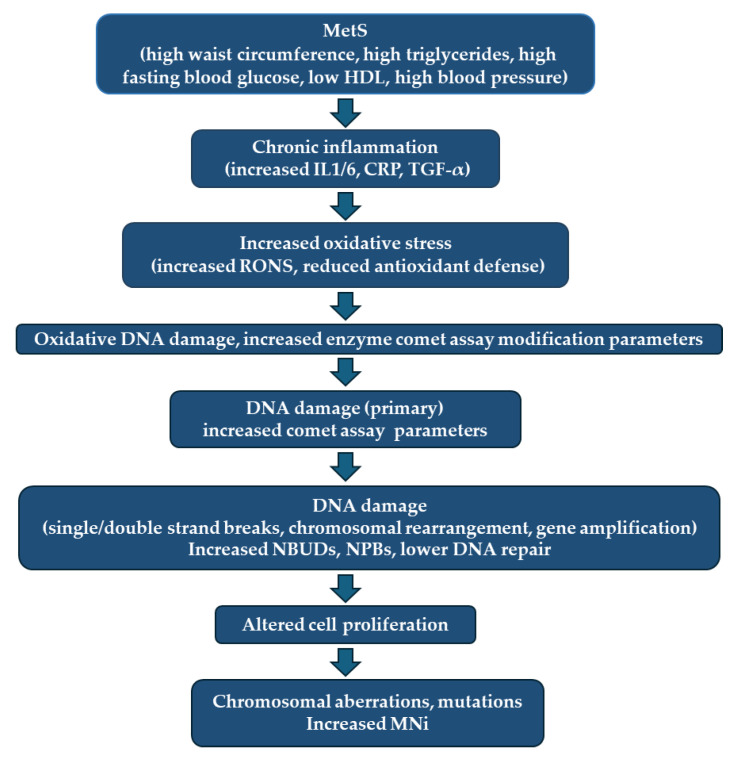
Link between metabolic syndrome and markers of CBMN, the comet assay, and its modifications (picture modified from Franzke et al., 2020 [31], and our findings [26]).

Based on the review article findings, particularly Franzke et al., 2020 [31], and our previous work [26], we provide a scheme of the association between metabolic syndrome, its severity, and the appearance of different types of DNA damage (see Figure 3).

Some authors have reported that, in normal-weight obese patients, elevated plasma cytokine levels are present and accompanied by an increase in some oxidative stress markers [69,70]. This phenomenon is not observed in patients with obesity, highlighting the processes that begin in normal-weight obesity before the onset of metabolic syndrome [60,61]. Fieres et al., 2022 [71], also found a significant linear association between higher BMI and higher levels of basal DNA damage. The impaired total DNA repair capacity was also observed in the PBMC of men within the normal-to-overweight range. This confirms again that reduced DNA repair capacity might be a potential mechanism underlying the DNA damage with a higher BMI.

Since primary prevention is a fundamental strategy for diminishing the overall burden of MetS, identifying biomarkers for early detection and prognosis is paramount to reduce the progression to T2D and CVD [26]. Franzke et al., 2020 [31], suggested that the MN assay and the frequency of different types of DNA damage measured by this assay should be used to establish not only baseline values but also individual baseline values for healthy populations. The use of all of these parameters should enable us to detect and define the critical levels of genome damage for disease risk assessment. It should also help distinguish how to further use the MN frequency as a marker to predict and evaluate the essential outcomes of disease. In our previous study on individuals with obesity, we demonstrated that individuals with severe obesity exhibit significantly elevated levels of DNA damage [26]. The highest levels of damage were observed among individuals with the highest BMI and weight. We also identified a subgroup of individuals with the highest levels of MN frequency who had a personal or family history of tumors and cancers [26].

Studies have shown that the level of DNA damage can decrease even with a modest weight loss over 12 weeks [38]. However, it is important to note that, even after dietary interventions, the DNA damage levels measured with both assays remain higher than the values generally accepted for healthy controls [26,43].

## 4. Conclusions and Future Directions

In conclusion, normal-weight obesity can set the stage for the onset of MetS, characterized by increased inflammation, oxidative damage, and reduced DNA repair capacity, which may contribute to the higher DNA damage seen with an elevated BMI. Excessive oxidative stress can cause elevated levels of DNA damage. While oxidative stress biomarkers for MetS detection are not highly specific due to their instability and short half-life in the bloodstream, they include biomarkers of lipid peroxidation, protein and amino acid oxidation, and DNA oxidation (for details and the specific names of potential oxidative stress biomarkers, refer to Masenga et al., 2023 [10]). Despite their lack of specificity, studies have demonstrated that the indirect detection of MetS is reliable, depending on certain molecules (lipids, proteins, and DNA) significantly affected by oxidative stress [10]. Combining these biomarkers and molecular targets can help develop innovative methods for preventing, diagnosing, treating inflammatory and metabolic disorders, and preventing comorbidities. Some studies have suggested pharmacotherapeutic and dietary approaches to address this, but there is a need to develop a set of sensitive biomarkers further. Both assays (MN with all of its parameters and comet with its modifications) used alongside other biomarkers in analyzing the MetS severity and disease development have proven valuable. These assays also demonstrate their utility in the prediction the progression from MetS to T2D and cardiovascular disease (CVD) in adults and children (for details, refer to Andreassi et al., 2011) [25]. However, future studies involving a larger number of patients and utilizing all of the parameters of the MN assay are necessary, as these types of studies have been limited. A comprehensive dataset is still needed to gain better insights into the additional potential of these assays. Additionally, these assays can help monitor prevention or therapy strategies, especially with GLP-1 agonists. These agonists have been shown to significantly improve anthropometric and metabolic parameters in patients with T2D and obesity [72], which could influence outcomes, and improve health stability and the results of both assays. However, this type of study is yet to be conducted. While cancer studies were not addressed here, it is well-established that both assays demonstrate elevated levels in such cases, and overweight/obesity is linked to an increased risk of at least 13 different cancers [62]. According to the IARC report from 2016, which analyzed over 1000 epidemiological studies, the absence of excess body fat lowers the risks of most cancers [62].

Given the ability of both assays to estimate cancer and mortality risks, we strongly recommend their inclusion in studies examining the severity of MetS and its complications. They could also serve as biomarkers in therapy and disease prevention efforts.

Using both assays in individuals may expand the prognostic power of established biomarkers for detecting and progressing from MetS to T2D and CVD in adults and children (for details, refer to Andreassi et al., 2011) [25]. Furthermore, utilizing both assays in the same individuals would help to clarify whether DNA damage acts as a biomarker or a mediator in MetS and its comorbidities, as an independent cardiometabolic risk factor, or merely as a surrogate for chronic oxidative stress and inflammation.

## Figures and Tables

**Table 1:** Diagnostic criteria and cut-off points of MetS according to various organizations (table modified from Rusell et al., 2024 and Alberti et al., 2009) [6,7].

Parameter/Criteria	Harmonised Criteria-2009	IDF-2005	NCEP ATP III-Revised Version 2001
Prerequisites for MetS diagnosis	Any three of the following	Abdominal obesity, along with any other two	Any three of the following
Waist circumference (cm)	Non-Europeans, IDF criteria Europeans, IDF or AHA/NHLBI cut-off points (102 cm for males and 88 for females)	Ethnic cut-off points	Ethnic-specific
Men	≥90 cm	≥94 cm (Asian ≥ 90 cm, WHO)	≥102 cm (Asian 90 cm, WHO)
Women	≥80 cm	≥80 cm	≥88 cm (Asian 80 cm, WHO)
HDL-C (mmol/L)	HDL-C treatment	HDL-C treatment	HDL-C treatment
	Or	Or	Or
Men < 40	<1.03	<1.03	<1.03
Women < 50	<1.30	<1.30	<1.30
Blood pressure (mmHg)	Hypertension treatment or previously diagnosed hypertension or	Hypertension treatment or previously diagnosed hypertension or	Hypertension treatment or previously diagnosed hypertension or
	systolic ≥ 130c diastolic ≥ 85	systolic ≥ 130 diastolic ≥ 85	systolic ≥ 130 diastolic ≥ 85
Fasting blood glucose (mmol/L)	Treatment or elevated glucose or previously diagnosed type 2 diabetes or	Treatment or elevated glucose or previously diagnosed type 2 diabetes or	Treatment or elevated glucose or previously diagnosed type 2 diabetes or
	≥5.6	≥5.6	≥5.6
Triglyceride (mmol/L)	TG treatment or ≥1.7 (150 mg/dL)	TG treatment or ≥1.7 (150 mg/dL)	TG treatment or ≥1.7 (150 mg/dL)

Different cut-off values for the MetS diagnosis are influenced by criteria that differ based on population- and country-specific definitions. The most widely used ones are those established and reviewed by the International Diabetes Federation (IDF) and the National Cholesterol Education Program Adult Treatment Panel III (NCEP ATP III) [6]. Differences in cut-off values based on these criteria are presented in Table 1, with further clarification provided in the harmonized criteria statement by several organizations, such as the International Diabetes Federation Task Force on Epidemiology and Prevention, National Heart, Lung, and Blood Institute (NHLBI), American Heart Association (AHA), World Heart Federation, International Atherosclerosis Society, and International Association for the Study of Obesity [6,7]. Part of this statement specifies that waist circumference should not be an obligatory component of MetS diagnosis. Instead, a diagnosis can be made with three abnormal findings, similar to the NCEP criteria. The IDF continues to maintain waist circumference as a required criterion, along with any two additional criteria from Table 1. Recent AHA/NHLBI guidelines for metabolic syndrome recognize an increased risk for CVD and diabetes at waist circumference thresholds of ≥94 cm in men and ≥80 cm in women, and identify these as optional cut-off points for individuals or populations with increased insulin resistance. WHO—World Health Organization; HDL-C—high-density lipoprotein cholesterol; TG—triglyceride.

## Data Availability

Data are available upon request.

## Supplementary Materials

The following supporting information can be downloaded at: https://www.mdpi.com/article/10.3390/medicina61010021/s1, The prisma checklist.
